# Methylome-wide analysis of IVF neonates that underwent embryo culture in different media revealed no significant differences

**DOI:** 10.1038/s41525-022-00310-3

**Published:** 2022-06-29

**Authors:** Rebekka M. Koeck, Florence Busato, Jorg Tost, Dimitri Consten, Jannie van Echten-Arends, Sebastiaan Mastenbroek, Yvonne Wurth, Sylvie Remy, Sabine Langie, Tim S. Nawrot, Michelle Plusquin, Rossella Alfano, Esmée M. Bijnens, Marij Gielen, Ron van Golde, John C. M. Dumoulin, Han Brunner, Aafke P. A. van Montfoort, Masoud Zamani Esteki

**Affiliations:** 1 Department of Clinical Genetics, Maastricht University Medical Centre+, Maastricht, The Netherlands; 2 Department of Genetics and Cell Biology, GROW School for Oncology and Reproduction, Maastricht University, Maastricht, The Netherlands; 3 Laboratory for Epigenetics & Environment, Centre National de Recherche en Genomique Humaine, CEA – institut de Biologie François Jacob, Université Paris Saclay, 91000 Evry, France; 4 Center for Reproductive Medicine, St. Elisabeth-TweeSteden Hospital, Hilvarenbeekseweg 60, 5022 GC Tilburg, the Netherlands; 5 Section of Reproductive Medicine, Department of Obstetrics and Gynecology, University Medical Center Groningen, University of Groningen, Hanzeplein 1, 9713 GZ Groningen, the Netherlands; 6 Center for Reproductive Medicine, Amsterdam Reproduction & Development Research Institute, Amsterdam UMC, University of Amsterdam, Meibergdreef 9, 1105 AZ Amsterdam, the Netherlands; 7 Health Unit, Flemish Institute for Technological Research (VITO), Boeretang 200, 2400 Mol, Belgium; 8 Department of Pharmacology & Toxicology, School for Nutrition and Translational Research in Metabolism (NUTRIM), Maastricht University, Maastricht, The Netherlands; 9 Centre for Environmental Sciences, Hasselt University, Diepenbeek, Belgium; 10 Department of Public Health and Primary Care, Leuven University (KU Leuven), Leuven, Belgium; 11 Department of Human Structure and Repair, Ghent University Hospital, Ghent, Belgium; 12 Department of Epidemiology and Nutrition and Toxicology Research Institute Maastricht (NUTRIM), Maastricht University Medical Centre, Maastricht, the Netherlands; 13 Department of Obstetrics and Gynaecology, GROW School for Oncology and Reproduction, Maastricht University Medical Center+, Maastricht, The Netherlands; 14 Department of Human Genetics, Radboud University Medical Center, Nijmegen, The Netherlands

**Keywords:** DNA methylation, Outcomes research

## Abstract

A growing number of children born are conceived through in vitro fertilisation (IVF), which has been linked to an increased risk of adverse perinatal outcomes, as well as altered growth profiles and cardiometabolic differences in the resultant individuals. Some of these outcomes have also been shown to be influenced by the use of different IVF culture media and this effect is hypothesised to be mediated epigenetically, e.g. through the methylome. As such, we profiled the umbilical cord blood methylome of IVF neonates that underwent preimplantation embryo development in two different IVF culture media (G5 or HTF), using the Infinium Human Methylation EPIC BeadChip. We found no significant methylation differences between the two groups in terms of: (i) systematic differences at CpG sites or regions, (ii) imprinted sites/genes or birth weight-associated sites, (iii) stochastic differences presenting as DNA methylation outliers or differentially variable sites, and (iv) epigenetic gestational age acceleration.

## Introduction

Since its first successful implementation in 1978, more than 8 million children^1^ (~3% of all births in European countries) have been conceived through in vitro fertilisation (IVF)^2^. Although most of these children are born seemingly healthy, assisted reproductive technology (ART) singletons are at increased risk of adverse perinatal^3^ and childhood^4,5^ outcomes as compared to their naturally conceived counterparts. For instance, IVF neonates are at higher risk of preterm birth (<37 weeks, relative risk (RR) 1.4–2.0), low birth weight (<2500 g, RR 1.6–1.7), being small for gestational age (RR 1.5) and perinatal mortality (RR 1.7–2.0)^3^. Later life outcomes mainly relate to growth and weight, as well as disturbed cardiometabolic function, demonstrated by increased systolic blood pressure, suboptimal diastolic function, lower low-density lipoprotein and higher fasting insulin levels^4–6^.

The IVF process involves 2–6 days of in vitro embryo culture, during which embryos are exposed to an artificial environment that is influenced by the culture medium, atmospheric conditions (oxygen levels) and laboratory plastics. Over the years, a variety of culture media have been used^7–11^, which have been shown to affect short- and long-term health outcomes of the resultant offspring in both animal and human studies. In human studies culture medium composition has been linked to differences in birth weight^12–14^, postnatal weight^15,16^ and the childhood developmental profile^17^. Previously, we conducted a multi-centre randomised controlled trial (RCT) among six Dutch IVF centres to compare the effect of G5 (Vitrolife) and HTF (Lonza) media on pregnancy and neonatal outcomes. Of note is that the G5 medium contains amino acids^8,18^, while HTF does not. While it was found that G5 led to lower fertilisation rates, it generated more embryos that were suitable for transfer and had a higher implantation rate, leading to a higher cumulative live birth rate^14^. At birth, G5 neonates were more likely to be born prematurely and with lower birth weights^14^ even when birth weight was corrected for gestational age, indicating an additional effect of the culture medium on birth weight.

Although no causative mechanism for these differences in outcome has been established, the findings are consistent with the Developmental Origins of Health and Disease (DOHaD) paradigm. This paradigm suggests that adversity during early life, such as during the peri-conception period, makes the resultant offspring more vulnerable to disease in later life^19^ and this effect may be mediated by the epigenome, and specifically DNA methylation^20^. In the context of IVF, the handling of gametes and embryos and exposure to the in vitro environment or the hormone-primed uterus represent environmental exposures that could contribute to the observed disease susceptibility^21^. Further evidence for the involvement of DNA methylation is that epigenetically regulated imprinting disorders, although still rare, are more common after IVF^22^. Moreover, the period of in vitro embryo culture of IVF procedures coincides with the process of epigenetic reprogramming, during which DNA methylation marks are almost completely erased and re-established^23,24^. This process has been shown to be responsive to environmental cues^24^.

Relatively few studies have used molecular assays to assess the effects of different IVF culture media on the resultant embryos and neonates. For instance, the methylome of IVF neonates from a culture medium trial has only been investigated in one prior study. As a follow-up to the aforementioned G5 versus HTF RCT, placental DNA methylation at selected imprinting control regions was compared in resultant singletons finding no significant differences within these regions^25^. In contrast, most other work so far has focused on comparing the placenta or umbilical cord blood (UCB) methylome of IVF neonates in general to their naturally conceived counterparts^26–32^. These studies were recently summarised in a systematic review and meta-analysis^30^ which described that most sites or regions identified to be differentially methylated were inconsistent or contradictory between studies, likely due to differences in the methylome analysis methods, heterogeneity within the cohorts and due to sample size. The majority of included studies used targeted approaches to look at imprinting genes, and a meta-analysis of such studies conducted on the placenta and UCB samples revealed only significant differential methylation at the *PEG1/MEST* imprinting gene locus^30^. Methylation at the imprinted regions *KvDMR1*, *H19* CTCF3 and CTCF6 and *SNRPN* may also be perturbed in IVF placentas, but these did not reach statistical significance in the meta-analysis^30^. The epigenetic deregulation in these cases is thought to occur post-fertilisation as it involves both paternally and maternally methylated regions and the methylation levels differ only by a few percent, indicating that the loss or gain of methylation only affects a minority of alleles. The findings from genome-wide methylation studies on these tissues have been contradictory, with some studies identifying differential methylation, predominantly with small differences, and others not^29–31^. Interestingly, some studies report increased variation in DNA methylation in IVF offspring^28,29^, suggesting a stochastic rather than a systematic universal effect of IVF on the methylome. This is substantiated by the reported increased rate of so-called methylation outliers (i.e. samples with an outlying methylation value at a given site or region) in the IVF group^25^. The contribution of different culture media to systematic or stochastic methylome differences on a genome-wide scale remains undetermined.

**Fig. 1:**
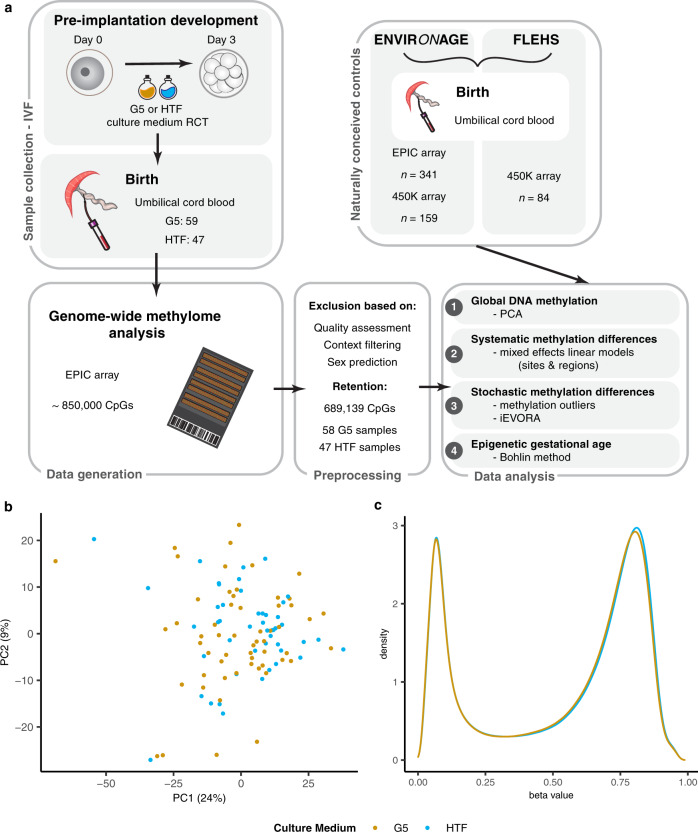
Genome-wide DNA methylation analysis of IVF neonates that underwent embryo culture in different media revealed no significant differences. **a** Schematic overview showing sample collection from IVF neonates from the G5 versus HTF RCT as well as the inclusion of naturally conceived neonates, genome-wide DNA methylation data generation, data processing and analyses included in this study. **b** PCA of all CpG sites passing our QC criteria in data from UCB samples of IVF neonates that underwent embryo culture in G5 (gold) or HTF (blue) medium. **c** Density plot showing the distribution of beta values from all sites and samples within each group (G5 = gold, HTF = blue).

In this study, we investigated the effect of different IVF culture media on the DNA methylation of human IVF neonates on a genome-wide scale. To this end, we profiled the UCB methylome of IVF neonates that underwent embryo culture in G5 or HTF medium as part of a RCT. Additionally, the methylome profiles of this IVF cohort are compared to data from two reference birth cohorts of naturally conceived individuals (Fig. 1a).

## Results

In the present study, we investigated genome-wide DNA methylation patterns of DNA samples derived from UCB collected at birth from 114 IVF neonates that had undergone embryo culture in G5 or HTF medium. 106 of the UCB samples (*n* = 59 G5, *n* = 47 HTF) yielded sufficient DNA for DNA methylation profiling using the EPIC array (Fig. 1a). Maternal characteristics, IVF treatment parameters and neonatal outcomes were comparable between the culture medium groups. In the G5 group, although not statistically significant, a higher percentage of pregnancies were complicated by hypertension and pre-eclampsia than in the HTF group (hypertension—14 vs. 6%, pre-eclampsia 7 vs. 2% for G5 and HTF pregnancies respectively). Delivery by caesarean section was lower (12%) in the G5 group compared to the HTF group (23%) (Table 1 and Supplementary Table 1).

**Table 1:** Maternal and neonatal characteristics (see also Supplementary Table 1).

Characteristic	Culture medium		*P* value
	G5 (*n* = 59)	HTF (*n* = 47)	
**Maternal characteristics**			
Age (years)	33.2 ± 3.6	33.1 ± 3.6	0.861
Nulliparous	43 (73)	35 (74)	1.000
Smoking before pregnancy (yes)	10 (17)	10 (21)	0.752
Smoking during pregnancy (yes)	2 (3)	3 (6)	0.794
**Fertility treatment**			
Indication for fertility treatment			0.630
Unexplained	8	8	
Female factor	14	8	
Male factor	35	31	
Treatment type			1.000
IVF	19 (33)	16 (34)	
ICSI	38 (67)	31 (66)	
**Pregnancy characteristics**			
Pregnancy complication			
Diabetes	2 (3)	1 (2)	1.000
Hypertension	8 (14)	3 (6)	0.362
Pre-eclampsia	4 (7)	1 (2)	0.496
Delivery by caesarean section	7 (12)	11 (23)	0.220
**Neonatal outcomes**			
Sex (female)	29 (49)	26 (55)	0.663
Gestational age at birth (weeks)	39.7 ± 1.2	39.3 ± 1.3	0.127
Birth weight (g)	3404.9 ± 459.7	3449.1 ± 432.5	0.672

Continuous variables are shown as mean ± SD and categorical variables are shown as *n* (%). Maternal age at the time of ovum pick-up is shown.

*ICSI* intracytoplasmic sperm injection.

**Fig. 2:**
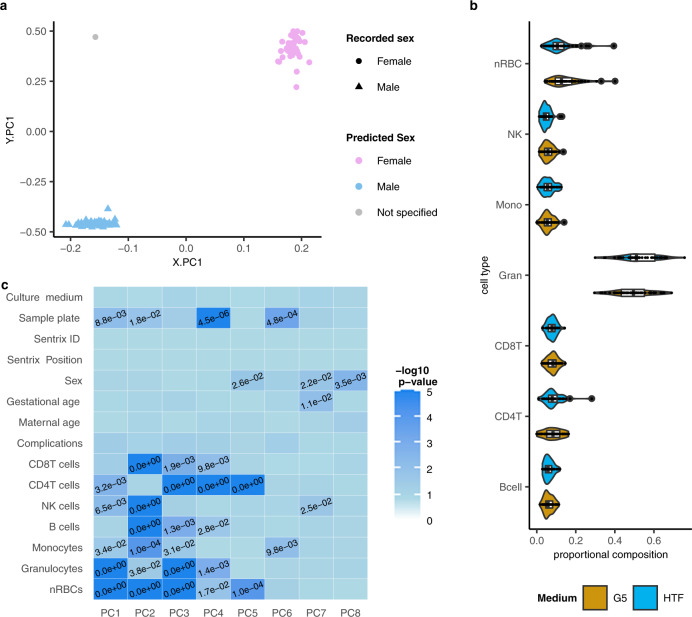
Preprocessing of umbilical cord blood (UCB) methylome data. **a** Scatter plot showing the projection of UCB samples (*n* = 105) into the PC space generated using reference data for sex prediction. The shape of the dots represents the recorded sex of the participants (circles = female, triangle = male), while the colour shows the predicted sex based on results from sEST (blue = male, pink = female, grey = not specified). **b** Violin plot showing the predicted cellular composition of the UCB samples, split by culture medium group (gold and blue represent G5 and HTF medium respectively). The violin plots are overlaid with boxplots where the horizontal lines represent the 25th percentile, median and 75th percentile respectively while the whiskers extend to the farthest data points that are no more than 1.5 times the IQR from the upper or lower quartile. **c** Heatmap showing associations between the principal components and biological/technical aspects of the samples. The colour gradient represents the −log10 of the *p* values. *P* values < 0.05 are shown. Significance of the correlation between age, maternal age, CD8-T cells, CD4T cells, NK cells, B cells, monocytes, granulocytes and nRBCs and the 8 PCs was tested using a permutation test with 10,000 permutations. The associations of the PCs with variables creating 2 groups (sample plate, sex, culture medium, pregnancy complication) and those creating three or more groups (Sentrix ID, Sentrix position) were tested using two-sided Wilcoxon rank tests and Kruskal–Wallis one-way analysis of variance respectively.

All of the 106 samples that underwent DNA methylation analysis by EPIC array met our QC criteria (Methods). One sample from the G5 group was excluded from our analyses based on a mismatch between the recorded and predicted sex (Fig. 2a). Of the approximately 850,000 CpG sites represented on the EPIC array, we retained 696,205 sites for our analyses and 689,139 of these represented complete observations with no missing values in any samples.

### Global analysis of DNA methylation

Principal component analysis (PCA) did not reveal any separation of the culture medium groups within the first eight principal components (PCs) (Figs. 1b, 2c) that explain a total of 46.7% of the variance within our data (Supplementary Table 2**)**, indicating that the culture media are not the main contributors to the variance of our data. Instead, the first eight PCs were significantly associated with sample characteristics including sex (PCs 5 and 8), gestational age (PC7), sample plate (PCs 1, 2, 4 and 6) as well as cellular composition of the samples (PCs 1–7) (Fig. 2b, c). Therefore, we corrected for these technical factors in our subsequent analyses, alongside potential confounders (sex, gestational age, maternal age, treatment centre and pregnancy complications) that were chosen a priori based on literature and expert opinion. The distribution of all beta values (all sites in all samples) was also similar between the culture medium groups (Fig. 1d).

### Analysis of DNA methylation at individual CpG sites

**Fig. 3:**
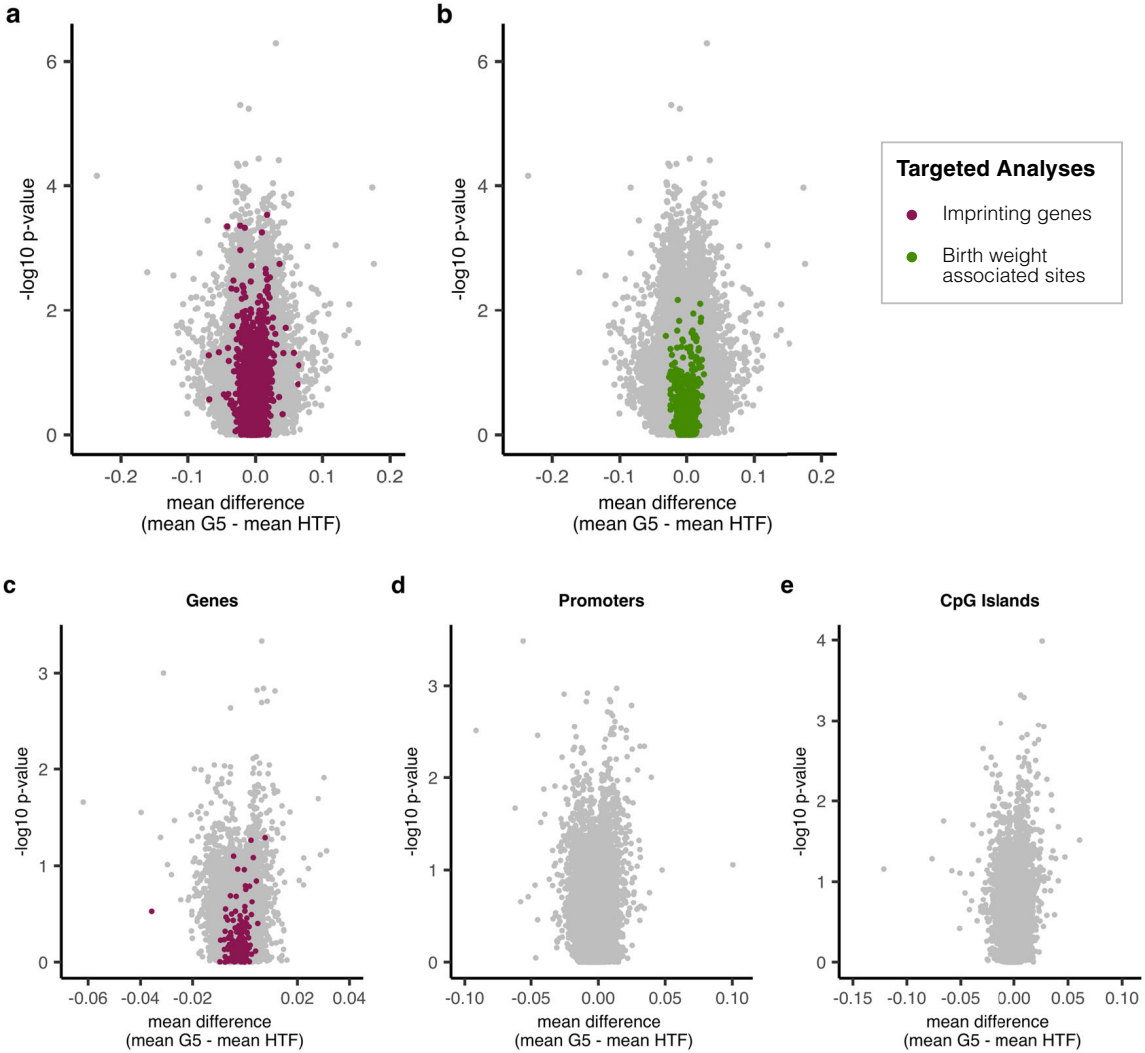
Analysis of systematic methylation differences between G5 and HTF neonates: differentially methylated positions and regions. Volcano plots showing differential methylation between G5 and HTF neonates where the grey dots represent all individual CpG sites (**a**, **b**) or multiple CpG sites aggregated into genomic regions, namely genes (**c**), promoters (**d**), CpG islands (**e**). Imprinted genes (**c**) and sites within them (**a**) are highlighted in purple while CpG sites associated with birth weight are shown in green (**b**). No significantly differentially methylation positions or regions (FDR adjusted *p* value < 0.1) were identified when comparing the two culture medium groups.

Next, we investigated associations between the culture medium and DNA methylation at single CpG sites in an epigenome-wide analysis (EWAS) using linear mixed-effects models (Methods). Less than 0.01% of sites (37 sites in total) had a group mean difference of more than 10%, with the most extreme difference being 23.6%. After correcting for multiple testing, no statistically significant differentially methylated positions (DMPs) were found between the two culture medium groups (Fig. 3a, b). As pregnancy complications, such as gestational diabetes and pre-eclampsia, could affect or be affected by the methylome, we conducted the analyses twice, once with pregnancy complications included as a binary variable (yes/no) and once where all samples from complicated pregnancies (*n* = 18) were excluded. The results from this analysis were comparable to those of the first analysis (Supplementary Fig. 1).

To reduce the number of comparisons, we also chose to repeat the analyses with sites of potential interest only, namely sites within imprinted genes^33^ and sites previously associated with birth weight^34^. After the data were pre-processed, 8726 sites within imprinted genes were tested. The maximum group mean difference amongst the imprinted sites was 6.9% (Fig. 3a). None of the sites were found to be significantly differentially methylated between the culture medium groups. Of the 914 CpG sites consistently found to be associated with birth weight in the meta-analysis by Küpers et al.^34^, 749 passed our quality control (QC) criteria and were included in the analysis. Amongst these sites, the maximal group mean difference was 3.0% and we did not find any of them to be statistically differentially methylated (Fig. 3b). Excluding the samples from complicated pregnancies did not change the result of either analysis (Supplementary Fig. 1a, b).

### Regional analysis of DNA methylation

After looking at the methylation levels of individual sites, we looked at methylation across larger genomic regions, namely whole genes, promoters, and CpG islands (CGIs). Our analyses included 28,009 genes, of which 207 were imprinted genes, 42,035 promoters and 25,238 CGIs. The maximal group mean difference of any gene was 8.1%. Imprinted genes showed even lower group mean differences than imprinted sites, with a maximal difference of 3.6%. No genes were found to be significantly differentially methylated between the G5 and HTF groups (Fig. 3c). The maximal group mean differences for promoters and CGIs were 10.0% and 12.1%, respectively (Fig. 3d, e) and no promoters or CGIs were found to be significantly differentially methylated between the culture medium groups. Excluding samples from pregnancies with complications did not affect the results (Supplementary Fig. 2a–c).

### Differential DNA methylation variance in IVF samples

**Fig. 4:**
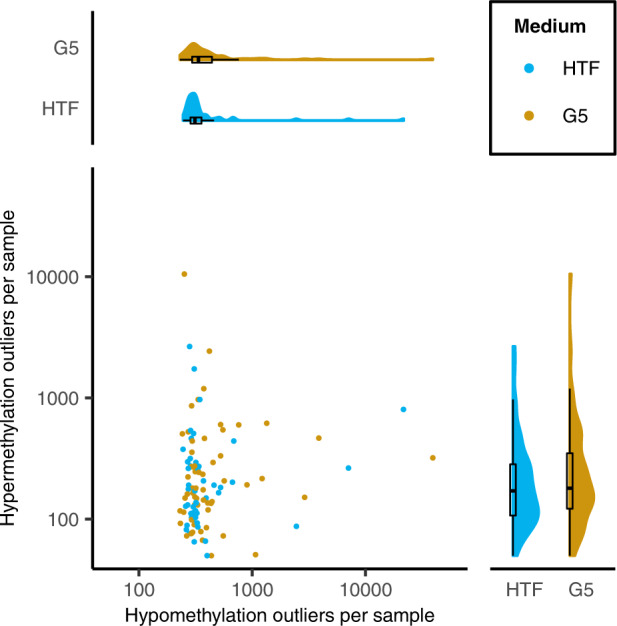
Methylation outliers. The main panel shows the number of hypomethylation (x-axis) and hypermethylation (y-axis) outliers per UCB sample (G5 = gold, HTF = blue). Distribution summaries, in the form of a density plot and boxplot, are shown for hypomethylation outliers and hypermethylation outliers in the top and right side panels respectively. Lines of the boxplot represent the 25th percentile, median and 75th percentile respectively while the whiskers extend to the farthest data point that is no more than 1.5 times the IQR from the upper or lower quartile. The axes are shown on a log10 scale. The groups were not found to be significantly different (*p* value > 0.1).

To assess the contribution of stochastic DNA methylation alterations to the observed phenotypes in our IVF cohorts, we assessed differential variance, using the iEVORA algorithm^35^, and identified methylation outliers, using previously described thresholds^36^, in all samples. Applying this threshold, we identified a total of 157,160 outliers within the 105 analysed samples, with a predominance of hypomethylation outliers (114,693 hypomethylation outliers and 42,467 hypermethylation outliers) (Fig. 4). The median number of all, both hypo- and hypermethylation, outliers in each G5 sample was 571 (567.5 IQR) and 536 (269 IQR) in each HTF sample (Fig. 4), which was not found to be significantly different (*p* = 0.86) between the culture medium groups. Furthermore, when considering hypomethylation and hypermethylation outliers separately, no significant difference was found between the culture medium groups. Outlier burden, the total number of outliers per sample, was not significantly associated with gestational age, birth weight or maternal age. Only technical features of our samples, including sample plate and cell composition, were significantly associated with outlier burden (Supplementary Table 3). An association between pregnancy complications and the total number of outliers was not tested statistically, but amongst the samples with very high numbers of outliers (above the upper quartile), only 1 was born after a pregnancy complicated by pre-eclampsia. The results were comparable when the samples taken from neonates that had experienced pregnancy complications were excluded (Supplementary Fig. 3 and Supplementary Table 4). When applied to the full cohort, the iEVORA algorithm identified 262 CpG sites with significantly different variances between the culture medium groups (Supplementary Table 5, sheet 1). Of these sites, 90% (235 sites) were more variable in the G5 group as compared to the HTF group. 202 of the 262 differentially variable CpG sites were annotated with a gene name and four genes, namely *FAM38A*, *MEF2C*, *OCA2* and *TNNT2,* each contained two differentially variable sites. Additionally, three of the differentially variable sites were located within imprinting genes, namely *PEX10*, *MAGI2* and *OBSCN*. None of the differentially variable sites were amongst the birth weight-associated sites^37^. We then repeated the analysis excluding all participants who had experienced pregnancy complications which identified 105 differentially variable sites (Supplementary Table 5, sheet 2). Of these sites, 56% (50 sites) were more variable in the G5 group than the HTF group and 65 of the sites were the same as those identified in the analysis where all the participants were included. Seventy-nine of the sites were annotated with a gene name and multiple differentially variable sites were identified in two of the genes, namely two sites within the *TNNT2* gene and three sites within the *MOV10L1* gene. Furthermore, one site was found to be differentially variable in the imprinted gene *PEX10* and none of the identified sites were birth weight-associated sites. GO and KEGG enrichment analyses of the differentially variable sites identified by iEVORA did not identify any significantly enriched ontologies or pathways after multiple testing corrections (Supplementary Table 5, sheets 4–7).

### Epigenetic gestational age as a marker of developmental maturity

**Fig. 5:**
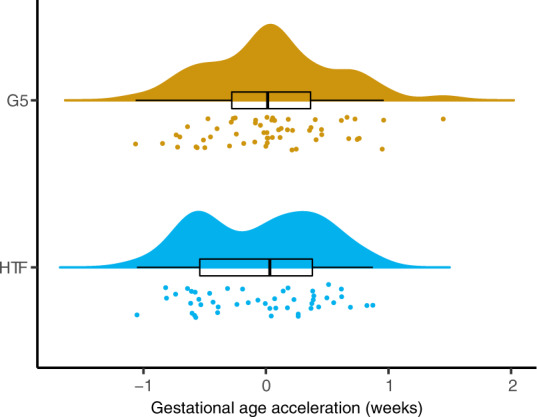
Epigenetic gestational age acceleration. Raincloud plot showing the GAA of each UCB sample in each culture medium group. Points represent individual samples of the G5 (gold) and HTF (blue) groups. Above a density plot and boxplot is shown. Horizontal lines of the boxplot represent the 25th percentile, median and 75th percentile respectively while the whiskers extend to the farthest data point that is no more than 1.5 times the IQR from the upper or lower quartile. GAA is represented in weeks. The groups were not found to be significantly different (*p* value > 0.1).

Gestational age can be predicted from DNA methylation levels at certain CpG sites (epigenetic clock)^38,39^. Similar to birth weight, these have been used to comment on developmental maturity at birth and gestational age acceleration (GAA), i.e. when epigenetic gestational age (eGA) is more advanced than clinical gestational age (cGA), has been positively correlated with birth weight^37,39,40^. eGA estimates derived using the Bohlin prediction model^38^ were more strongly correlated (Pearson correlation coefficient = 0.77) with our data and had a lower root mean squared error (RMSE = 1.29) than the estimates derived with the Knight prediction model^39^ (Pearson correlation coefficient = 0.55, RMSE = 1.45), therefore only the results from the Bohlin epigenetic clock are shown. However, of note is that both prediction models were trained using data from the HumanMethylation450 (450K) array and of the 96 sites used for the Bohlin eGA prediction model, eight sites with coefficients ranging from −15.5 to 6.1 are no longer present on the EPIC array. We removed these sites from the prediction model. When applying the prediction model to 450K data from the ENVIR*ON*AGE study (*n* = 159), the omission of these eight CpG sites lead to a mean increase in the predicted gestational age by 0.73 weeks (range 0.17–1.10 weeks) (Supplementary Fig. 4a). Therefore, we cannot thoroughly evaluate absolute epigenetic gestational age, but we assume that all samples will be similarly affected by the missing sites and thus can compare the GAA between culture medium groups. GAA was calculated by regressing eGA on cGA while correcting for cell composition of the samples (Methods). In G5 samples the median GAA was 0.01 (0.64 IQR) and in HTF samples the median GAA was 0.03 (0.92 IQR), which was not significantly different (*p* = 0.42) (Fig. 5). Additionally, we found no significant correlation between GAA and birth weight (Pearson correlation = −0.17, *p* = 0.08). The results were comparable when participants who had experienced pregnancy complications were excluded from the analysis (Supplementary Fig. 4b).

### Comparison of IVF neonates to naturally conceived neonates

Even though the main aim of this study was to investigate the effect of two different culture media on the methylome of IVF neonates, we also sought to compare the methylomes of the IVF neonates to those of naturally conceived neonates using previously published data from the FLEHS and ENVIR*ON*AGE longitudinal cohort studies. However, as these samples were not processed concurrently with the IVF samples it is not possible to correct for technical variation between the studies meaning that any effect of the IVF process cannot be differentiated from technical differences. These findings are demonstrated in the supplementary material (Supplementary Table 6: participant demographics, Supplementary Fig. 5: processing of FLEHS and ENVIR*ON*AGE data, Supplementary Fig. 6: comparison of IVF and naturally conceived neonates).

## Discussion

To the best of our knowledge, the genome-wide analysis of the influence of different IVF culture media on the methylome of human IVF neonates presented here is the largest cohort on which such a study has been conducted to date. Despite this, our sample size was insufficient to conduct sub-group analyses looking specifically at sex or treatment type (IVF vs. ICSI), which could reveal clinically relevant differences. We have investigated the impact of two compositionally different media, namely G5 from Vitrolife and HTF from Lonza, which were shown to influence IVF outcomes during the original RCT^14^. However, these phenotypic differences, e.g. in birth weight, were no longer significant in the sub-group of the original RCT that is presented here. We have found no evidence that these culture media lead to systematic or stochastic methylation differences in the resultant IVF neonates. To facilitate a comparison between different modes of conception, samples from well-matched naturally conceived individuals would have ideally been collected and processed alongside the IVF samples.

In line with findings from previous studies, examining the methylome of IVF children born after embryo culture in different media, we identified no differentially methylated positions or regions and only moderate group mean differences, largely less than 10%. This was also seen when the methylation status of imprinting genes in the placenta of the same individuals was analysed^25^. Similarly, a comparison of IVF children (aged 7 or 8) born after embryo culture in a global medium (Life Global), or single-step medium (Irvine Scientific) found no evidence of differential methylation between the medium groups at imprinting genes, transposable elements or on a genome-wide scale^41,42^.

The lack of differential methylation between G5 and HTF neonates may seem surprising given the stark differences in medium composition, which include the complete lack of amino acids in HTF medium, while G5 contains all amino acids except non-essential glutamate, glutamine and glycine^8^, and the addition of hyaluronan and lipoic acid to G5 medium^14^. Although the direct interplay between these individual components and DNA methylation has not been investigated in human embryos, it seems plausible that amino acid availability may influence the functional capacity of DNA methylation establishment and maintenance machinery. The sensitivity of embryos to these environmental differences is further supported by the finding that gene expression differences exist between embryos cultured in G5 or HTF medium^43,44^ and it is known that gene expression can be regulated by DNA methylation. However, the lack of differentially methylated sites or regions could be explained by a number of reasons. Firstly, dysregulated DNA methylation may be transient during in vitro embryogenesis and therefore not be detectable in neonates. Secondly, alternative epigenetic marks, such as histone modifications, may mediate the association between the culture media and the observed gene expression and phenotype differences. Additionally, in the sub-group of participants recruited for this follow-up study, phenotypic differences, such as birth weight, were less than in the full RCT cohort, which may have reduced the magnitude of any culture medium-induced effects. Finally, even though our study is the largest described methylome study after an IVF culture medium trial, we still lack the power to detect methylation differences with a magnitude of less than 10%. Although exact power estimates are challenging without the existence of prior data to establish the expected variance within our study population, simulation studies by Saffari et al.^45^ and Tsai et al.^46^ estimates that a sample size of 211 or more participants would be required to achieve 80% power to detect significant methylation differences with an effect size of 7% or less, respectively, using array-based assays such as the EPIC array^45,46^. However, it remains to be determined whether mean differences of less than 10%, representing methylation loss or gain at any site in just a small proportion of an individual’s cells, represent clinically significant differences^47^.

An alternative to the theory that peri-conception environmental differences induce systematic methylation differences, relates to the presence of stochastic epimutations that are either induced by the environment^48^ or provide a survival benefit if selection pressure is applied by certain environmental conditions^49^. In placenta samples of the same individuals as those described in this study, DNA methylation outliers were also identified in all samples without a difference in outlier burden between the culture medium groups^25^. Whether the number of outliers identified per individual is comparable between the two studies is difficult to assess due to the different thresholds used to define outliers and the different techniques used to analyse the methylome that differ vastly in their coverage of the genome. In the field of cancer biology, the iEVORA algorithm has been used to identify so-called field defects, which represent stochastic methylation alterations in normal pre-cancerous tissues that later undergo neoplastic transformation^35^. Frequently, sites identified as differentially variable in pre-cancerous samples become differentially methylated in tumour samples, suggesting that sites of this nature could be interesting biomarkers for disease with a later onset. In this study, such epimutations could be linked to later development of disease phenotypes, such as cardiometabolic diseases, although it should be noted that the differentially variable sites identified were not enriched in pathways relating to cardiovascular or metabolic function and it is not yet known whether there will be a difference in the prevalence of cardiometabolic disease between G5 and HTF offspring. Nonetheless, these sites warrant further clinical and molecular follow-up. Alternatively, differential variability at certain CpG sites could be driven by factors that are only experienced by a few individuals in the study population, such as pregnancy complications. Previously, DNA methylation differences associated with pre-eclampsia^50^ and gestational diabetes^51–53^ have been described when analysing UCB samples of neonates. According to our findings, there might be an association between culture medium, the number of differentially variable sites and pregnancy complications, but the design of this study does not allow to discuss the direction of causality (culture media, methylation and pregnancy complications).

Although studies comparing naturally conceived and IVF neonates have found some methylation differences, especially at imprinting genes^30^, this has not been observed in this or other culture medium comparisons^25,41,42^. This may be due to the fact that the environmental discrepancy between two culture media is less severe than the difference between in vivo and in vitro embryo development, thus leading to a smaller or no effect on the methylome. The concurrent processing of samples from naturally conceived individuals would be required to assess this further.

The lack of difference we observed in GAA may relate to the fact that the eGA prediction tools were trained using the HumanMethylation450K array and eight of the 96 probes required for the prediction model are no longer present on the EPIC array. These probes were therefore excluded from the model leading to a consistent over-estimation of gestational age. The inclusion of these sites may be important to identify a relationship between GAA, birth weight^37,39,40^ and potentially culture medium.

In conclusion, our genome-wide methylome analysis of IVF neonates that underwent embryo culture in G5 or HTF medium revealed no significant differences between the culture medium groups, suggesting that the use of either culture medium will establish a comparable DNA methylation signature, including at imprinting genes. However, we have observed some differentially variable sites between the culture medium groups, which seem associated with pregnancy complications, but the persistence and clinical significance of these findings should be assessed with further follow-up studies. To assess whether epigenetic reprogramming is transiently affected by differences in culture medium composition, epigenetic studies of embryos cultured in different media are required.

## Methods

### Ethical approval

This study was approved by the local medical ethical committee, Medische Ethische Commissie academisch ziekenhuis Maastricht/University of Maastricht (METC azM/UM) and registered in the Dutch Trial register (NTR 1979/NL1866). Both parents of all neonates gave written informed consent.

### Study population and sample collection

Samples were collected as part of a culture medium comparison study^14^, which was a multi-centre RCT, involving six IVF centres in the Netherlands. Specifically, couples undergoing IVF treatments were randomised to embryo culture either in HTF medium (Lonza, Verviers, Belgium) or Vitrolife G1^TM^ Version 5 (G5, Göteborg, Sweden), while all other IVF-related procedures and conditions were kept the same. Of the 6 IVF centres, five participated in UCB sampling. In these five centres, the study resulted in 273 singleton live births that occurred after fresh (not frozen) embryo transfers. UCB samples were collected from as many resulting singleton pregnancies as possible, 115 in total, irrespective of birth weight, gestational age at birth and the presence of pregnancy complications. Within 30 min of delivery, UCB was collected by a gynaecologist, nurse or midwife according to a standardised protocol. The samples were sent to the Department of Obstetrics and Gynaecology at Maastricht University Medical Centre (MUMC+) and were stored at −80 °C until they were used.

### DNA extraction

DNA was extracted from thawed UCB samples using the Gentra Puregene DNA purification kit (Qiagen Hilden, Germany) according to the manufacturer’s instructions for 3 mL of human whole blood with minor modifications, namely, a smaller volume (8.5 mL) of red blood cell (RBC) lysis solution and longer centrifugation time (4 min where 2 min are indicated and 8 min where 5 min are indicated).

### Bisulfite conversion and methylome profiling by EPIC array

One microgram of DNA was bisulfite-treated using the EpiTect^®^ Fast 96 DNA Bisulfite Kit (Qiagen Hilden, Germany) and analysed using the Infinium Human Methylation EPIC BeadChip Kit (Illumina, CA, USA) according to the manufacturer’s protocol.

### Data analysis

All data were analysed using R (version 3.6.3)^54^. The data were visualised using the ggplot2^55^ and ComplexHeatmap^56^ packages.

#### Baseline characteristics

Differences in baseline characteristics between the two culture medium groups were compared and evaluated using Student’s *t*-tests for continuous variables and Pearson’s chi-squared tests for categorical variables.

#### Quality control and preprocessing

We applied preprocessing functions from the RnBeads package^57^ to normalise the data using subset-quantile within array normalisation (SWAN)^58^, and to remove poor quality probes and samples using the greedycut algorithm with a detection *p* value threshold of 0.05. Subsequently, the following sites were removed: (i) sites on the sex chromosomes, (ii) sites in close proximity to single nucleotide polymorphisms (SNPs), (iii) sites with missing values in more than 5% of the samples, and (iv) sites not in a CpG context. Sites containing missing values in 0–5% of the samples were only used to calculate aggregated beta values for different regions, including genes, promoters and CpG islands (CGI), they were excluded in all analyses looking at individual sites. Unless indicated otherwise, we used methylation beta values, which are calculated for each individual, at each CpG site by dividing the methylated signal intensity by the sum of the methylated and unmethylated signal intensity. The sex of the samples was predicted by comparing the samples’ sex chromosome methylation values and their respective detection *p* values to reference data using a clustering (PCA)-based approach, as implemented in the sEst package^59^. Correspondingly, each sample was assigned two predicted sexes based on the X-chromosome and Y-chromosome profiles respectively. If both matched, the sample was labelled as male or female, otherwise, it was labelled as “not specified”. If a mismatch between the recorded and predicted sex was identified, the sample was removed from subsequent analyses (*n* = 1).

#### Cellular deconvolution of UCB samples

To estimate the cellular composition of the UCB samples, the reference-based method described by Gervin et al.^60^ was implemented using the minfi package^61^. As recommended, the algorithm was applied to data pre-processed with the *preprocessNoob* method^62^ and deconvolution was carried out based on the IDOL optimised probes contained within the FlowSorted.CordBloodCombined.450k package^63^. The algorithm was used to estimate the proportion of natural killer cells, B cells, monocytes, granulocytes, nucleated red blood cells and CD4- and CD8-T cells within each sample.

#### Comparison of G5 and HTF IVF neonates

All high-quality CpG sites were used to conduct a PCA in which the beta values were centred but not scaled. Associations between the PCs and technical or demographic features of the samples were tested using: (i) permutation tests (with 10,000 permutations) to ascertain the significance of correlations (gestational age, maternal age, predicted cellular sample composition), (ii) a two-sided Wilcoxon rank test for categorical data where there are two groups (sex, culture medium, sample plate, pregnancy complication) or (iii) a Kruskal–Wallis one-way analysis of variance for categorical variables generating 3 or more groups (Sentrix ID, Sentrix position—array number and sample position respectively).

Methylation M-values, representing the log2 ratio of the methylated probe intensity compared to the unmethylated probe intensity^64^, were used to test for an association between the culture medium and DNA methylation with mixed-effects linear models implemented using the variancePartition package^65^. The models were corrected for potential confounders, namely gestational age, sex, maternal age, pregnancy complications (included as a binary variable where the presence of gestational diabetes, hypertension and pre-eclampsia were encoded as “yes” and otherwise “no” was recorded) and the predicted cell compositions as fixed effects while the treatment centre and batch effects (sample plate) were included as random effects. As gestational diabetes, hypertension and pre-eclampsia represent pathophysiologically heterogeneous pregnancy complications, the analyses were repeated while excluding participants affected by any of the complications. The models were applied to individual CpG sites or aggregate (mean) values of multiple CpG sites within a region to identify differentially methylated positions (DMPs) or regions (DMRs) respectively. To examine DMRs, the M-values of all probes attributed to a specific gene, promoter or CGI were aggregated by calculating their mean. For the targeted analyses, the models described above were applied to (sites within) imprinted genes^33^ and probes associated with birth weight^34^. All analyses were corrected for multiple testing using the Benjamini–Hochberg method^66^, and an adjusted *p* value of <0.1 was considered significant.

DNA methylation outliers were defined as described previously^36^. In short, hypomethylation outliers were defined as beta values lower than three interquartile ranges (IQR) from the 25th percentile, while hypermethylation outliers were defined as beta values greater than three times the IQR above the 75th percentile. The IQR and percentile values were calculated using all UCB samples. Subsequently, an association between the log_10_ transformed number of outliers and the culture medium was sought using the mixed-effects linear models as described above. To identify CpG sites with differential variance between the culture medium groups, we applied iEVORA^35^ using the matrixTests package^67^. At each CpG site, iEVORA applies Bartlett’s test, which is a parametric test for differential variance, as well as a Student’s *t*-test. Thereafter, sites reaching significance in Bartlett’s test after multiple testing correction (FDR corrected *p* value < 0.05) and nominal significance in the t-test (*p* value < 0.05 without multiple testing correction) are considered significant. As such, the output of Bartlett’s test is regularised as it is usually overly sensitive to single outliers. Both DNA methylation outliers and iEVORA analyses were applied to the full cohort as well as the subset of participants that had not experienced pregnancy complications. Gene ontology (GO) and Kyoto Encyclopaedia of Genes and Genomes (KEGG) pathway enrichment analyses were conducted on differentially variable sites using functionality from the missMethyl package^68^.

#### Estimation of epigenetic gestational age and gestational age acceleration

Epigenetic gestational age was calculated using methods described by Bohlin et al.^38^ and Knight et al.^39^. The accuracy of the respective predictions was evaluated by calculating the Pearson’s correlation and root mean squared error between eGA and cGA. The model described by Bohlin et al. generated more accurate predictions and was therefore used to calculate GAA as previously described^38^. The Bohlin eGA prediction model was applied exactly as described by Bohlin et al. Firstly, within array normalisation was carried out using the BMIQ method using the RnBeads package^57^. Subsequently, batch effects attributable to the sample plate were corrected using ComBat from the sva package^69^. There were no missing values in any samples at the required sites, apart from eight CpG sites of the prediction model that are not present on the EPIC array. These eight sites were therefore excluded from the prediction. GAA represents the residuals from regressing eGA on cGA corrected for sample cell composition. To determine whether there is an association between GAA and culture medium, we applied mixed-effects linear models correcting for sex and maternal age as fixed effects alongside IVF treatment centre as a random effect. Again, the analysis was carried out on the full cohort and repeated excluding those participants who had experienced pregnancy complications.

#### Comparison of IVF and naturally conceived neonates

##### Selection and processing of data from naturally conceived individuals

To compare the methylome of our IVF neonates to naturally conceived individuals, we used data from two geographically similar longitudinal birth cohorts, namely the Flemish Environment and Health Study (FLEHS, Flanders Belgium)^70,71^ and the Environmental Influence on Early Ageing study (ENVIR*ON*AGE)^72^ that had both undertaken array-based methylome profiling. Samples were considered for inclusion if the neonates were born after at least 36 full weeks of gestation (comparable to the IVF neonates included in this study). A total of 85 individuals from the FLEHS cohort and 502 individuals from the ENIR*ON*AGE cohort were considered for inclusion based on this criterium. Methylation data in these studies were generated either with Illumina’s EPIC or 450K arrays. Preprocessing of the data from the separate studies/arrays was conducted separately but in an identical fashion to the IVF data, with the exception of within-study batch effects, which were corrected using ComBat^69^ as these could not be corrected for using the mixed-effects models. The sample inclusion and preprocessing steps of these two cohorts is summarised in Supplementary Fig. 5H. After study/data type-specific processing the data were combined, retaining only CpG sites present and passing the QC of all the array types included. Overall, 346,403 CpG sites were common to all platforms and studies.

To select only the data likely to be most similar to our IVF cohort, we generated a matched selection from the ENIR*ON*AGE neonates who had their methylome profiled using the EPIC array. We used nearest neighbour matching (Mahalanobis distance) based on sex, maternal age, birth weight and gestational age to select 105 neonates to compare to the IVF neonates. This matching was carried out using the MatchIt package^73^.

##### Comparison of characteristics of matched IVF and naturally conceived individuals

The participant characteristics between IVF and matched naturally conceived individuals were compared and evaluated using Student’s *t*-tests for continuous variables and Pearson’s chi-squared tests for categorical variables. The *p* values obtained from this comparison are shown in Supplementary Table 6.

##### Statistical testing to compare naturally conceived and IVF neonates

Empirical Bayes moderated mixed effect linear models were used to ascertain associations between DNA methylation and mode of conception. These models were corrected for gestational age at birth, cell composition, sex, and maternal age as fixed effects and where relevant, array type as a random effect. Multiple testing correction was applied using the Benjamini–Hochberg method^66^, and an adjusted *p* value of <0.05 was considered significant.

### Reporting Summary

Further information on research design is available in the Nature Research Reporting Summary linked to this article.

## Supplementary information

Supplementary material

Supplementary table 5

Reporting Summary Checklist

## Data Availability

The dataset generated during the current study, IVF samples, are available in the Gene Expression Omnibus (GEO) repository^74^ under the accession number GSE189531. Data included from the FLEHS and ENVIR*ON*AGE cohorts are available from GEO under the accession numbers GSE110128 and GSE151042 respectively.

## Code availability

Custom R code used for the processing and analysis of the data described in this article are available at https://github.com/CellularGenomicMedicine/UCB_methylome.
