# Supplementary material for: Methylome-wide analysis of IVF neonates that underwent embryo culture in different media revealed no significant differences
Source: NPJ Genom Med. 2022 Jun 29;7:39. doi: 10.1038/s41525-022-00310-3 (PMC9243125; doi:10.1038/s41525-022-00310-3)
Supplement: Supplementary file 1 — Supplementary material [file 41525_2022_310_MOESM1_ESM.pdf]

SUPPLEMENTARY INFORMATION

Methylome-wide analysis of IVF neonates that underwent embryo culture in different media revealed no significant differences

Rebekka M. Koeck[1, 2], Florence Busato[3], Jorg Tost[3], Dimitri Consten[4], Jannie van Echten-Arends[5], Sebastiaan Mastenbroek[6], Yvonne Wurth[4], Sylvie Remy[7], Sabine Langie[7, 8], Tim S. Nawrot[9, 10], Michelle Plusquin[9], Rossella Alfano[9], Esmée M. Bijnens[9, 11], Marij Gielen[12], Ron van Golde[13], John C.M. Dumoulin[13], Han Brunner[1, 14], Aafke P.A. van Montfoort[13*], Masoud Zamani Esteki[1, 2*]

Affiliations

1. Department of Clinical Genetics, Maastricht University Medical Centre+, Maastricht, The Netherlands
2. Department of Genetics and Cell Biology, GROW School for Oncology and Reproduction, Maastricht University, Maastricht, The Netherlands
3. Laboratory for Epigenetics & Environment, Centre National de Recherche en Genomique Humaine, CEA – institut de Biologie François Jacob Université Paris Saclay, 91000 Evry, France
4. Center for Reproductive Medicine, St. Elisabeth-TweeSteden Hospital, Hilvarenbeekseweg 60, 5022 GC, Tilburg, the Netherlands.
5. Section of Reproductive Medicine, Department of Obstetrics and Gynecology, University Medical Center Groningen, University of Groningen, Hanzeplein 1, 9713 GZ, Groningen, the Netherlands.
6. Center for Reproductive Medicine, Amsterdam Reproduction & Development Research Institute, Amsterdam UMC, University of Amsterdam, Meibergdreef 9, 1105 AZ Amsterdam, the Netherlands
7. Health Unit, Flemish Institute for Technological Research (VITO), Boeretang 200, 2400, Mol, Belgium
8. Department of Pharmacology & Toxicology, School for Nutrition and Translational Research in Metabolism (NUTRIM), Maastricht University, The Netherlands
9. Centre for Environmental Sciences, Hasselt University, Diepenbeek, Belgium
10. Department of Public Health and Primary Care, Leuven University (KU Leuven), Leuven, Belgium
11. Department of Human Structure and Repair, Ghent University Hospital, Ghent, Belgium
12. Department of Epidemiology and Nutrition and Toxicology Research Institute Maastricht (NUTRIM), Maastricht University Medical Centre, Maastricht, the Netherlands
13. Department of Obstetrics and Gynaecology, GROW School for Oncology and Reproduction, Maastricht University Medical Center+, Maastricht, The Netherlands
14. Department of Human Genetics, Radboud University Medical Center, Nijmegen, The Netherlands

* These authors jointly supervised this study

Corresponding authors: Aafke P.A. van Montfoort: aafke.van.montfoort@mumc.nl and Masoud Zamani Esteki: masoud.zamaniesteki@mumc.nl

SUPPLEMENTARY FIGURES 3

SUPPLEMENTARY FIGURE 1| DNA METHYLATION AT INDIVIDUAL CPG SITES IN PARTICIPANTS WITHOUT PREGNANCY COMPLICATION. 3

SUPPLEMENTARY FIGURE 2| REGIONAL DNA METHYLATION IN PARTICIPANTS WITHOUT PREGNANCY COMPLICATIONS. 4

SUPPLEMENTARY FIGURE 3| METHYLATION OUTLIERS IN SAMPLES WITHOUT PREGNANCY COMPLICATIONS 5

SUPPLEMENTARY FIGURE 4| EPIGENETIC GESTATIONAL AGE ACCELERATION 6

SUPPLEMENTARY FIGURE 5| PROCESSING OF UCB METHYLOME DATA FROM NATURALLY CONCEIVED NEONATES FROM THE FLEHS AND ENVIRONAGE COHORTS 7

SUPPLEMENTARY FIGURE 6| COMPARISON OF IVF AND NATURALLY CONCEIVED INDIVIDUALS 9

SUPPLEMENTARY TABLES 11

SUPPLEMENTARY TABLE 1 | EXTENDED CHARACTERISTICS. 11

SUPPLEMENTARY TABLE 2 | VARIANCE EXPLAINED BY PCS 1-8 OF GLOBAL METHYLATION PCA ANALYSIS 12

SUPPLEMENTARY TABLE 3 | ASSOCIATION BETWEEN NUMBER OF OUTLIERS AND SAMPLE FEATURES. 13

SUPPLEMENTARY TABLE 4 | ASSOCIATION BETWEEN NUMBER OF OUTLIERS AND SAMPLE FEATURES IN PARTICIPANTS WITHOUT PREGNANCY COMPLICATIONS 14

SUPPLEMENTARY TABLE 5 | DIFFERENTIALLY VARIABLE SITES IDENTIFIED BY IEVORA (SEE THE EXCEL FILE IN THE ONLINE VERSION OF THE ARTICLE). 15

SUPPLEMENTARY TABLE 6 | CHARACTERISTICS OF NATURALLY CONCEIVED AND IVF NEONATES 16

Supplementary figures

Supplementary figure 1| DNA methylation at individual CpG sites in participants without pregnancy complication

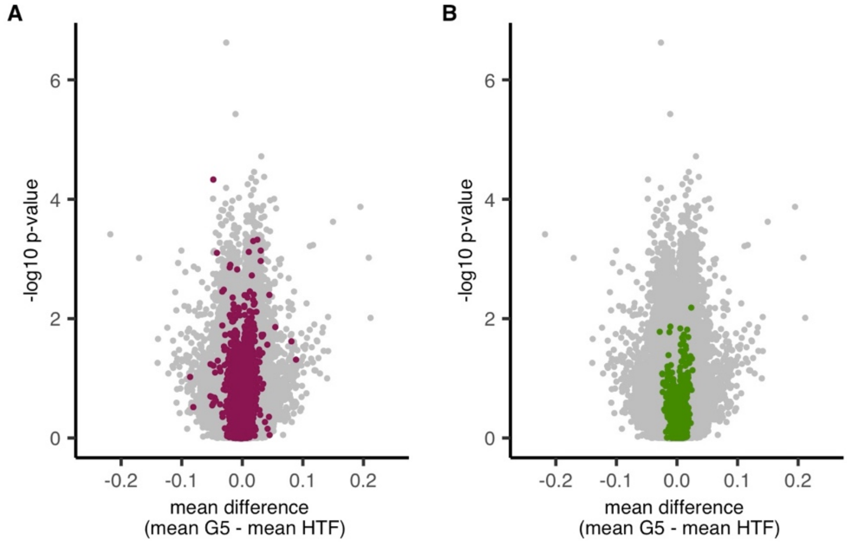

Volcano plots showing differential methylation between G5 and HTF neonates at all individual CpG sites (grey dots, **A**&**B**). Samples from neonates whose were pregnancies complicated by gestational diabetes, hypertension or preeclampsia were excluded. Highlighted in purple are CpG sites within imprinted genes (**A**) and in green are birth weight associated CpG sites (**B**). No sites were significantly differentially methylated (false discovery rate (FDR) adjusted p-value <0.1) between the culture medium groups.

Supplementary figure 2| Regional DNA methylation in participants without pregnancy complications

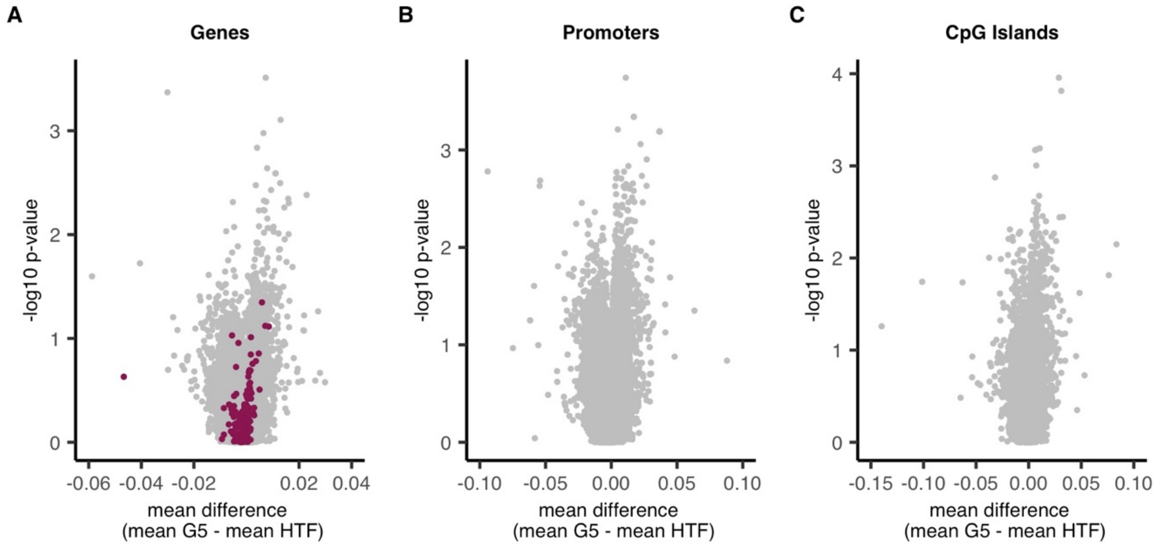

Volcano plots showing differential methylation between G5 and HTF neonates at multiple sites aggregated into regions: by gene (**A**), by allocation to distinct promoters (**B**) and by allocation to distinct CGIs (**C**). Samples from neonates whose pregnancies were complicated by gestational diabetes, hypertension or preeclampsia were excluded. Highlighted in purple are imprinted genes (**A**). No regions were significantly differentially methylated (false discovery rate (FDR) adjusted p-value <0.1) between the culture medium groups.

Supplementary figure 3| Methylation outliers in samples without pregnancy complications

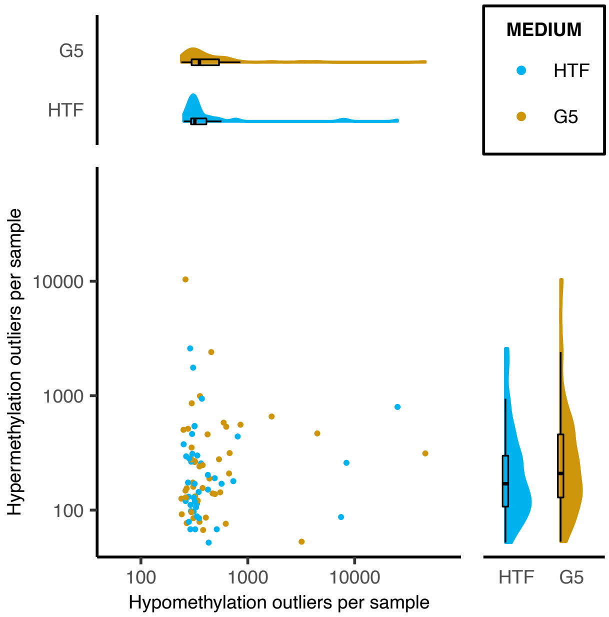

The main panel shows the number of hypomethylation (x-axis) and hypermethylation (y-axis) outliers per UCB sample (G5 = gold, HTF = blue). Distribution summaries, in the form of a density plot and boxplot, are shown for hypomethylation outliers and hypermethylation outliers in the top and right side-panels, respectively. Lines of the boxplot represent the 25th percentile, median and 75th percentile, respectively while the whiskers extend to the farthest data point that is no more than 1.5 times the IQR from the upper or lower quartile. The axes are shown on a log10 scale. The groups were not found to be significantly different (p-value >0.1).

Supplementary figure 4| Epigenetic gestational age acceleration

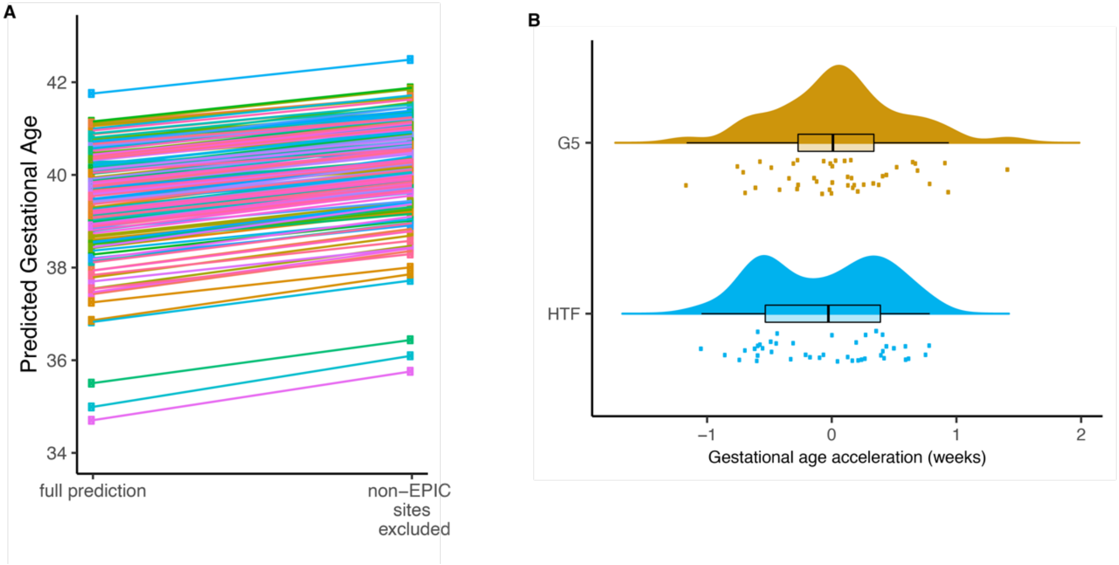

(**A**) Prediction of epigenetic gestational age using the Bohlin method on 450K data from the ENVIR*ON*AGE cohort (*n* = 159). Data points on the left show the prediction including all the specified sites, points on the right represent the predictions from the same samples when the 8 CpG sites that are not present on the EPIC array are excluded. (**B**) Raincloud plot showing the total number of outliers per umbilical cord blood (UCB) sample in each culture medium group when participants with pregnancy complications were excluded. Points represent individual samples of the G5 (gold) and HTF (blue) group. Above a density plot and boxplot is shown. Horizontal lines of the boxplot represent that 25th percentile, median and 75th percentile, respectively while the whiskers extend to the farthest data point that is no more than 1.5 times the IQR from the upper or lower quartile. GAA is represented in weeks. The groups were not found to be significantly different (p-value >0.1).

Supplementary figure 5| Processing of UCB methylome data from naturally conceived neonates from the FLEHS and ENVIRONAGE cohorts

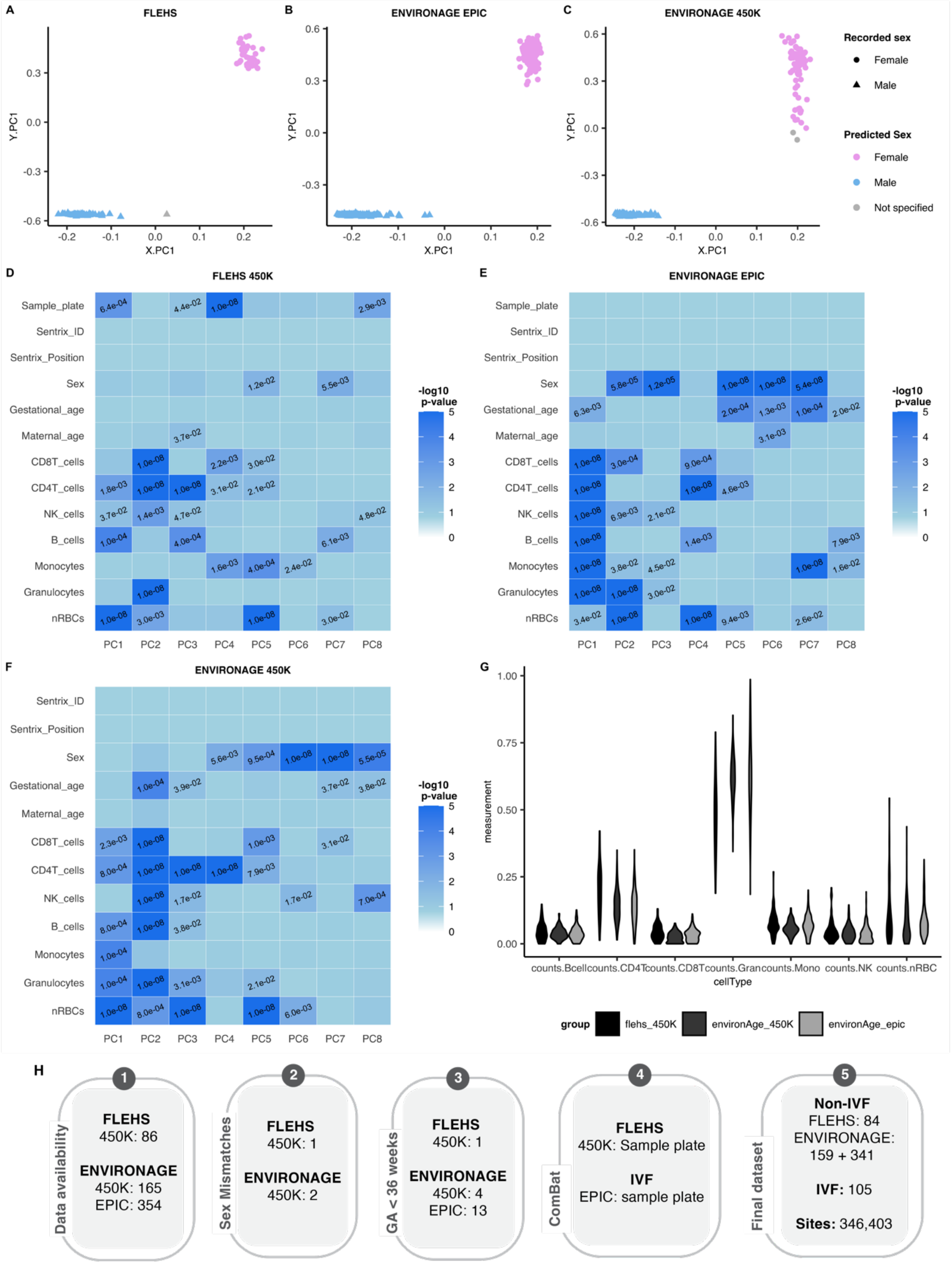

(**A**-**C**) Scatter plots showing the projection of UCB samples (FLEHS 450K (**A**), ENVIR*ON*AGE 450K (**B**), ENVIR*ON*AGE EPIC (**C**)) into the principal component (PC) space generated using reference data for sex prediction. The shape of the dots represents the recorded sex of the participants (circles = female, triangle = male), while the colour shows the predicted sex based on results from sEST (blue = male, pink = female, grey = not specified). (**D**-**F**) Heatmaps showing associations between the principal components and biological/technical aspects of the samples (FLEHS 450K (**D**), ENVIRONAGE 450K (**E**), ENVIRONAGE EPIC (**F**)). The colour gradient represents the -log10 of the p-values. P-values that are <0.05 are shown. Significance of the correlation between continuous variables and the 8 principal components (PCs) was tested using a permutation test with 10,000 permutations. The associations of the PCs with variables creating 2 groups and those creating 3 or more groups were tested using two-sided Wilcoxon rank tests and Kruskall-Wallis one-way analysis of variance, respectively. (**G**) Violin plot showing the predicted cellular composition of the UCB samples, split by cohort and array type. (**H**) Overview of the data processing procedure showing the number of samples from each cohort excluded at each step, the batch effects that were pre-corrected using ComBat and the data included in the final dataset.

Supplementary figure 6| Comparison of IVF and naturally conceived individuals

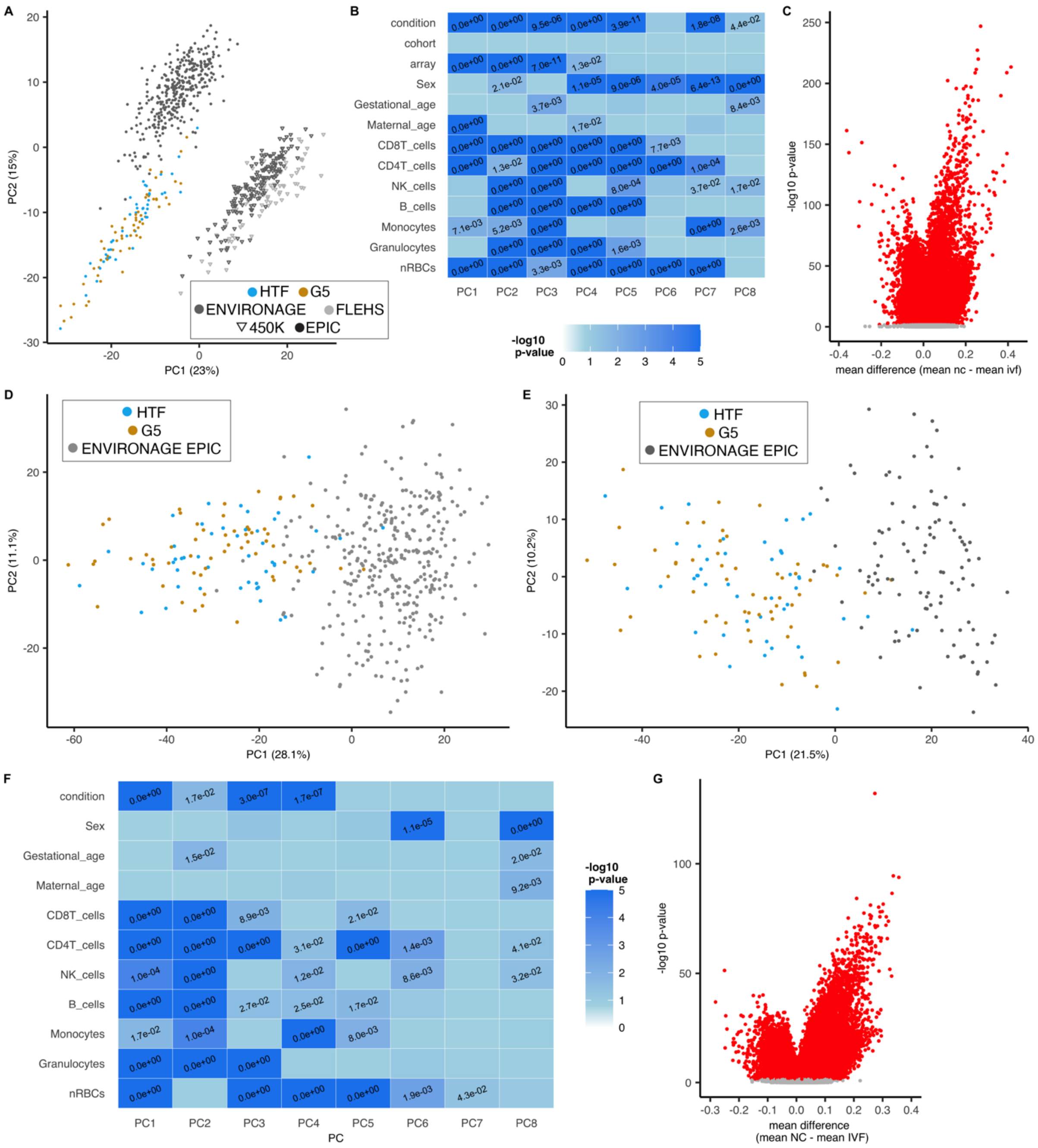

(**A**) PCA including CpG sites that are present on the EPIC and 450K array and passed the QC criteria in all three studies that analysed the methylome of UCB samples: IVF study (G5 = gold, HTF = blue) and two studies that collected samples from naturally conceived neonates (ENVIR*ON*AGE = dark grey, FLEHS = light grey). The shapes represent the array used to profile the samples (EPIC = dots, 450K = triangles). (**B**) Heatmap showing associations between the principal components and biological/technical aspects of the samples shown in **A** (*n* = 584 naturally conceived, *n* = 105 IVF). (**C**) Volcano plots showing differential methylation between naturally conceived (*n* = 584) and IVF neonates (*n* = 105) at individual CpG sites (*n* = 346,403). (**D**) PCA including high quality CpG sites with available data both in IVF (*n* = 105, blue and gold) and ENVIR*ON*AGE EPIC (*n* = 341, grey) samples. (**E**) PCA including high quality CpG sites with available data in IVF (*n* = 105, blue and gold) and matched ENVIR*ON*AGE EPIC (*n* = 105, grey) samples. (**F**) Heatmap showing associations between the principal components and biological/technical aspects of the samples shown in **E** (*n* = 105 naturally conceived, *n* = 105 IVF). (**G**) Volcano plot

showing differential methylation between IVF (*n* = 105) and matched naturally conceived (*n* = 105) neonates from the ENVIR*ON*AGE study analysed with the EPIC array. Individual CpG sites (*n* = 671,145) are shown. All PCA plots show the first two PCs, with the variance explained by each shown in brackets. In the heatmaps the colour gradient represents the -log10 of the p-values. P-values that are <0.05 are shown. Significance of the correlation between continuous variables and the 8 principal components (PCs) was tested using a permutation test with 10,000 permutations. The associations of the PCs with variables creating 2 groups and those creating 3 or more groups were tested using two-sided Wilcoxon rank tests and Kruskall-Wallis one-way analysis of variance, respectively. Volcano plots show significantly differentially methylated sites/regions (FDR adjusted p-value <0.05) in red and all other sites/regions in grey.

Supplementary Tables

Supplementary table 1 | Extended characteristics

Characteristic	Culture Medium		P-value
	G5 (*n* = 59)	HTF (*n* = 47)	
**Maternal characteristics**			
**Pre-pregnancy BMI**	23.8 ± 3.5	24.5 ± 4.3	0.371
**Paternal characteristics**			
**Age (years)**	33.1 ± 3.6	33.1 ± 3.7	0.995
**BMI**	25.4 ± 3.3	26.0 ± 3.0	0.295
**Fertility treatment**			
**IVF treatment centre**			0.659
**Amsterdam**	4	5	
**Groningen**	6	5	
**Maastricht**	26	23	
**Tilburg**	9	3	
**Eindhoven**	14	11	
**Egg donation (yes)**	0	0	1.000
**Sperm donation (yes)**	1	0	1.000

Continuous variables shown as mean ± SD, categorical variables shown as counts (number of individuals).

Supplementary table 2 | Variance explained by PCs 1-8 of global methylation PCA analysis

**PC**	**Variance explained (%)**
1	24.3
2	8.5
3	5.0
4	3.2
5	1.8
6	1.5
7	1.2
8	1.2
**Total:**	46.7

Supplementary table 3 | Association between number of outliers and sample features

	Hypomethylation outliers		Hypermethylation outliers		Total number of outliers	
**Feature**	**Correlation**	**Significance**	**Correlation**	**Significance**	**Correlation**	**Significance**
**Gestational age**	-0.03	0.772	0.07	0.511	-0.01	0.900
**Birth weight**	0.01	0.942	-0.07	0.462	-0.01	0.911
**Maternal age**	0.03	0.783	-0.06	0.517	0.01	0.913
**CD8T cells**	**0.24**	**0.014**	0.12	0.120	**0.26**	**0.007**
**CD4T cells**	-0.13	0.167	0.03	0.756	-0.12	0.206
**B cells**	0.18	0.060	0.18	0.060	0.22	**0.019**
**NK cells**	**0.33**	**0.002**	**0.20**	**0.049**	**0.37**	**0.001**
**Monocytes**	0.17	0.067	0.06	0.567	0.19	0.054
**Granulocytes**	**-0.36**	**0.000**	-0.15	0.119	**-0.39**	**0.000**
**nRBCs**	0.05	0.567	-0.04	0.693	0.04	0.639
**Sample plate***		**0.000**		**0.597**		**0.007**

NK = natural killer, nRBCs = nucleated red blood cells. Correlations shown are Pearson correlations and significance was tested with permutation tests (1000 permutation)

* significance tested with two-sided Wilcoxon rank test.

Supplementary table 4 | Association between number of outliers and sample features in participants without pregnancy complications

	Hypomethylation outliers		Hypermethylation outliers		Total number of outliers	
**Feature**	**Correlation**	**Significance**	**Correlation**	**Significance**	**Correlation**	**Significance**
**Gestational age**	-0.03	0.75	0.05	0.65	-0.02	0.83
**Birth weight**	-0.01	0.90	-0.10	0.38	-0.03	0.77
**Maternal age**	0.04	0.71	-0.07	0.49	0.02	0.82
**CD8T cells**	**0.24**	**0.03**	0.12	0.26	**0.26**	**0.02**
**CD4T cells**	-0.16	0.10	0.04	0.70	-0.15	0.14
**B cells**	0.18	0.09	0.19	0.06	**0.22**	**0.03**
**NK cells**	**0.36**	**0.00**	**0.23**	**0.04**	**0.40**	**0.00**
**Monocytes**	0.17	0.11	0.04	0.72	0.17	0.10
**Granulocytes**	**-0.40**	**0.00**	-0.19	0.06	**-0.43**	**0.00**
**nRBCs**	0.13	0.19	-0.00	1.00	0.13	0.20
**Sample plate***		**0.00**		0.73		**0.01**

NK = natural killer, nRBCs = nucleated red blood cells. Correlations shown are Pearson correlations and significance was tested with permutation tests (1000 permutation)

* significance tested with two-sided Wilcoxon rank test.

Supplementary Table 5 | Differentially variable sites identified by iEVORA *(see the excel file in the online version of the article)*

Differentially variable sites between the culture medium groups as identified by the iEVORA algorithm when applied to the full cohort (**Table sheet 1**) and when applied only on samples without pregnancy complications (**Table sheet 2**). Sites identified by both analyses are shown in **Table sheet 3**. Nominally significant GO enrichments for the iEVORA sites from the full cohort and the samples without pregnancy complications are shown in **Table sheet 4** and **Table sheet 5**, respectively. Nominally significantly enriched KEGG pathways for the iEVORA sites from the full cohort and the samples without pregnancy complications are shown in **Table sheet 6** and **Table sheet 7**, respectively.

Supplementary Table 6 | Characteristics of naturally conceived and IVF neonates

	**FLEHS**	**ENVIRONAGE**			**IVF**	
	**450K**	**450K**	**EPIC**	**Matched EPIC**	**G5 & HTF**	**Matched IVF-EPIC**
**Characteristic**	**(*n* = 84)**	**(*n* = 159)**	**(*n* = 341)**	**(*n* = 105)**	**(*n* = 105)**	**p-value**
**Maternal characteristics**						
**Age (years)**	28.8 ± 3.8	29.5 ± 4.5	30.2 ± 4.3	32.3 ± 3.5	33.1 ± 3.6	0.477
**Nulliparous**	54 (64)	80 (50)	179 (52)	46 (43.8)	77 (73.3)	**2.8e^{-06-}**
**Pregnancy characteristics**						
**Pregnancy complication**						0.851
**Diabetes**	NA	4 (3)	16 (5)	10 (9.5)	3 (2.9)	
**Hypertension**	NA	13 (8)	10 (3)	4 (3.8)	10 (9.5)	
**Preeclampsia**	NA	3 (2)	4 (1)	2 (1.9)	5 (4.8)	
**Neonatal outcomes**						
**Sex (female)**	39 (46)	75 (47)	179 (52)	54 (51.4)	54 (51.4)	1.000
**Gestational age at birth (completed weeks)**	39.3 ± 1.1	39.3 ± 1.2	39.3 ± 1.2	39.2 ± 1.2	39.2 ± 1.3	0.957
**Birth weight (g)**	3391 ± 459	3433 ± 477	3430 ± 425	3425 ± 416	3426 ± 448	0.982

Continuous variables shown as mean ± SD, categorical variables shown as n (%).
